# Improved developmental potential of mouse vitrified‐warmed oocytes achieved by culturing in recovery medium with glutathione ethyl ester (GSH‐OEt)

**DOI:** 10.1002/rmb2.12411

**Published:** 2021-08-27

**Authors:** Yoshihisa Harada, Masayuki Kinutani, Toshitaka Horiuchi

**Affiliations:** ^1^ Kinutani Women's Clinic Hiroshima Japan; ^2^ Emeritus Professor of Prefectural University of Hiroshima Hiroshima Japan; ^3^ Institute for Advanced Reproductive Medicine Ochi Yume Clinic Nagoya Nagoya Japan

**Keywords:** glutathione, intracytoplasmic sperm injection, oocyte vitrification, reactive oxygen species, time‐lapse imaging

## Abstract

**Purpose:**

The aim of the present study was to investigate the effect of glutathione ethyl ester (GSH‐OEt) in the recovery medium on the developmental competence of mouse vitrified‐warmed MII oocytes.

**Methods:**

Vitrified‐warmed oocytes were incubated for 1 h in recovery medium in the presence or absence of 0.5 mM GSH‐OEt. The authors examined the effects of GSH‐OEt, first on the levels of glutathione (GSH) and reactive oxygen species (ROS) in vitrified‐warmed oocytes, and second, on in vitro blastocyst development, division speed to blastocysts, and total cell numbers of blastocysts from vitrified‐warmed oocytes fertilized by Intracytoplasmic sperm injection (ICSI).

**Results:**

Adding GSH‐OEt to the recovery medium significantly (*p* < 0.05) increased GSH content and decreased ROS levels in vitrified‐warmed oocytes. The blastocyst rate did not differ significantly between the two groups, but the speed of development to blastocysts in the GSH‐OEt (+) group was significantly more rapid. In addition, the total blastocyst cell number was significantly higher in the GSH‐OEt (+) group than in the GSH‐OEt (−) group (92.8 ± 5.1 vs. 71.4 ± 3.5, *p* < 0.01).

**Conclusion:**

Adding GSH‐OEt to the recovery medium of vitrified‐warmed mouse oocytes enhances the development potential of oocytes and improves the quality of blastocysts.

## 1 INTRODUCTION

Cryopreservation of oocytes and embryos has been an essential technique in human assisted reproductive technology (ART). Since the first successful cryopreservation of mouse embryos was achieved with a slow‐freezing method in 1972,^1^ human embryos were first successfully cryopreserved with the same method by Trounson and Mohr in 1983 and obtained live birth.^2^ A vitrification cryopreservation method was first reported by Rall and Fahy in 1985, and this method was an ice‐free cryopreservation procedure with rapid cooling.^3^ The efficacy of vitrification using a rapid cooling rate and minimal fluid volume is demonstrated by the high survival rates of human embryos and oocytes when the cryotop method is used.^4^, ^5^, ^6^, ^7^, ^8^ Especially, oocyte cryopreservation is increasingly used in Japan, for social and oncofertility reasons.

On the other hand, the vitrification method requires the exposure of oocytes to a high concentration of cryoprotectants and increased osmolalities.^9^, ^10^, ^11^ So that, most intracellular water is replaced by a cell membrane‐permeable cryoprotectant. In particular, unfertilized oocytes are likely to be damaged by vitrification.^12^ The reason is that there is no change in the oocyte membrane structure that occurs during fertilization, unlike in the zygote. Oocytes are difficult cells to cryopreserve, due to their low surface area to volume ratio and high susceptibility to intracellular ice formation.^13^ The developmental potential and implantation capacity of fertilized embryos derived from vitrified oocytes are clearly lower than those of fresh oocytes.^14^, ^15^

Cryopreservation by vitrification is associated with an increase in reactive oxygen species (ROS) in oocytes.^16^, ^17^ Generally, the increase in ROS in cells is erased by glutathione (GSH)‐related GSH/GSSG redox potential.^18^ Alterations in GSH‐related GSH/GSSG redox potential induce post‐ovulatory aging, compromise male pronuclear formation, and cause embryo development to fail.^19^, ^20^ GSH is synthesized during oocyte maturation and reaches its maximum concentration before sperm penetration.^21^ Thus, GSH is synthesized via through the γ‐glutamyl cycle. Even if GSH is added extracellularly, it cannot penetrate the cell membrane and does not act directly on the oocyte.^18^ Glutathione ethyl ester (GSH‐OEt) easily penetrates the cell membrane and undergoes hydrolysis by intracellular esterase thereby increasing intracellular GSH concentrations.^22^, ^23^

Previous studies with bovine oocytes have reported that the addition of GSH‐OEt is effective against ROS production by long‐term in vitro cultures such as in IVM.^24^ Trapphoff et al^25^ reported that supplementation of media with GSH‐OEt immediately prior to vitrification of IVM oocytes prevents from oxidative damage and improves freezing damage. Furthermore, in vitrification of MII mouse oocytes, supplementation of GSH‐OEt immediately before vitrification was effective to embryo development.^26^

These studies show the effectiveness of pre‐vitrification GSH‐OEt supplementation, but the direct improvement of GSH content in oocytes after vitrification‐warming is expected rather than pre‐vitrification. Therefore, this study focused on effectiveness of adding GSH‐OEt to the recovery medium post‐vitrification and warming. To our knowledge, no studies have asked whether there is an additive effect of GSH‐OEt in the recovery medium of vitrified‐warmed oocytes on their in vitro development into blastocysts and on the quality of the blastocysts.

The purpose of this study was to investigate the additive effect of GSH‐OEt in the recovery culture medium of vitrified‐warmed mouse oocytes on embryo development after ICSI. Blastocyst quality was evaluated using time‐lapse analysis as non‐invasive assessment.^27^, ^28^

## 2 MATERIALS AND METHODS

### 2.1 Animals

In this study, we used B6D2F1 female mice, 3–4 weeks of age, obtained from CLEA Japan Inc. (Tokyo Japan). All mice were maintained in a temperature‐ and light‐controlled room at 25°C, with a 12‐h light/12‐h dark cycle (light starting at 6:00 h). These experiments were approved by the Committee for Ethics in Animal Experiments at the Prefectural University of Hiroshima, Japan (18A010), and were performed at Prefecture University of Hiroshima from June 2018 to March 2019.

### 2.2 Collection of oocytes

Female mice were superovulated by intraperitoneal injection of 5 IU pregnant mare serum gonadotropin (Serotropin; Asuka Pharmaceuticals), followed 48 h later by intraperitoneal injection of 5 IU human chorionic gonadotropin (hCG; Gonadotropin, Asuka Pharmaceuticals). At approximately 15 h post hCG injection, cumulus‐oocyte complexes (COCs) were collected from the oviduct. The collected COCs were denuded by pipetting with 250 IU/ml hyaluronidase within 5 min. We assessed denuded oocyte maturity based on the presence of the first polar body. The MII oocytes were cultured in a humid incubator at 37.0°C under 6% CO_2,_ 5% O_2_, and 89% N_2_ for approximately 30 min, until vitrification.

### 2.3 Vitrification and warming of mouse oocytes, and recovery culture

Oocytes were equilibrated in equilibration solution containing 7.5% (v/v) ethylene glycol (EG; Sigma‐Aldrich), 7.5% (v/v) dimethyl sulfoxide (DMSO; Nacalai Tesque Inc., Kyoto, Japan) in modified human tubal fluid (mHTF; Irvine Scientific), supplemented with 20% (v/v) SSS (Serum Substitute Supplement; Irvine Scientific) for 15 min at 25–28°C. After equilibration, the oocytes were transferred to vitrification solution containing 15% (v/v) EG, 15% (v/v) DMSO, and 0.5 M sucrose (Sigma‐Aldrich) in mHTF supplemented with 20% (v/v) SSS for 1.5 min at 25–28°C. Ten oocytes were placed in cryotops (Kitazato Supply; Fujinomiya, Japan) in a minimum volume, and then immediately plunged into liquid nitrogen. Vitrified oocytes were warmed sequentially in three solutions (1.0, 0.5, and 0.25 M sucrose) supplemented with 20% (v/v) SSS. The oocyte warming procedure was performed by immersing the tip of the cryotop directly into a 1.0 M sucrose solution for 1 min at 37°C. Next, the oocytes were transferred to a 0.5 M sucrose solution for 3 min and then to a 0.25 M sucrose solution for 5 min. Oocytes were then washed once in mHTF supplemented with SSS for 5 min at 37°C. After warming, the oocytes were incubated in global total medium supplemented with three different concentrations (0.5, 1.0, and 3.0 mM) of GSH‐OEt for 1 h.

### 2.4 Sperm collection and freezing

Spermatozoa were collected from the cauda epididymis of B6D2F1 mice at 12–16 weeks of age. As previously reported, for sperm freezing and sonication, we used an EGTA solution containing 10 mM Tris‐HCl buffer with 50 mM ethylene glycol‐bis (‐aminoethyl ether)—N, N, N, N—tetraacetic acid (EGTA solution).^29^, ^30^ Spermatozoa were suspended in approximately 1 ml of EGTA solution for up to 5 min at 37.0°C under 5% CO_2_ in air. Spermatozoa were centrifuged at 500 *g* for 5 min, the supernatant was removed, and the spermatozoa were mixed with 1 ml of EGTA solution. This suspension was transferred to ice water (0°C), then sonicated for 5 s at 50% sonicator output (20 kHz, VP‐5S; Taitec, Japan). More than 90% of mouse spermatozoa underwent head and tail separation. Finally, 100‐μl aliquots of sonicated spermatozoa in EGTA solution were placed in 0.25 ml cryostraws and frozen in liquid nitrogen (−196°C).

### 2.5 Microinjection of sperm heads into oocytes

Piezo‐ICSI was performed using a microscope (ECLIPSE Ti‐U; Nikon, Tokyo, Japan), equipped with a micromanipulator (Narishige Inc., Tokyo, Japan) and a piezo impact drive (MB‐U; Prime tech Ltd., Japan). The piezo drive unit was driven by a controller (PMAS‐ET150; Prime Tech Ltd., Ibaraki, Japan). Injection pipettes had an inner diameter of 5.95 μm and an outer diameter of 7 μm. One drop (15 μl) of frozen‐thawed sonicated sperm heads was suspended in 10% polyvinylpyrrolidone (PVP: Irvine Scientific) at a 1:1 ratio before injection. The MII oocytes were placed into 5 μl droplets of mHTF that contained 20% SSS, which had been placed next to sperm head‐containing droplets (15 μl) covered with mineral oil (Irvine Scientific). The zona pellucida of each oocyte was penetrated by applying several piezo pulses (speed 2, intensity 2), and the oolemma was then broken by applying a single piezo pulse (speed 1, intensity 1). All procedures were performed at room temperature. The piezo‐ICSI procedure was completed within 1 h of sperm thawing.

### 2.6 Measurement of GSH and ROS in vitrified‐warmed oocytes

Intracellular reduced GSH was analyzed using 4‐chloromethyl‐6,8‐difluoro‐7‐hydroxycoumarin (Cell Tracker Blue; Molecular Probes, Eugene, OR, USA). Ten oocytes from each group were incubated in the dark in 10 μM CellTracker Blue for 30 min at 37°C. The oocytes were rinsed twice with PBS (−) containing 0.1% (w/v) polyvinyl alcohol (PVA). Oocyte GSH levels were measured using an inverted fluorescence microscope with UV filter (Blue image, 370 nm, BZ‐710, Keyence).

Intracellular ROS levels were measured using 2′7′‐dichlorodihydrofluorescein diacetate (CM‐H_2_DCFDA; Molecular Probes, Eugene, OR, USA). Ten oocytes from each group were incubated in the dark in 10 μM CM‐H_2_DCFDA for 30 min at 37°C. The oocytes were rinsed twice with PBS (−) containing 0.1% (w/v) polyvinyl alcohol (PVA). To analyze ROS levels, 2,7‐dichlorofluorescein (DCF) fluorescence signals were detected using an inverted fluorescence microscope (green channel: 480 nm, BZ‐X710, Keyence).

In measuring ROS and GSH levels, the average fluorescence intensity of fresh oocytes was set to 1.0, and the reported values represent fold differences in fluorescence intensity.

### 2.7 Assessment of blastocyst cell numbers

Blastocysts at the end of culture at 96 h were individually stained for total cell number. The blastocysts were rinsed twice with PBS (−), then fixed in ethanol and stained with Hoechst 33342 (20 µg/ml) for 5 min at room temperature. Total blastocyst cell numbers were determined using a fluorescence microscope.

### 2.8 Time‐lapse imaging and annotations

Embryos were individually cultured in an incubator equipped with a time‐lapse imaging system (Primo Vision; Vitrolife, Denmark) for 96 h, from the ICSI procedure up to the blastocyst stage. Oocytes were cultured in global total (Life Global; Guelph, ON, Canada) at 37.0°C under 6% CO_2,_ 5% O_2_, and 89% N_2_. Each embryo was cultured in a 100 μl drop of global total medium in a culture dish with 16 microwells. Images of the embryos were recorded automatically every 10 min. The time‐lapse analysis was evaluated up to the blastocyst stage as predictive of blastocyst quality. Data were collected for the following time points: the time to second polar body appearance (2PB), the time to male and female pronuclei appearance (PNa), the time to male and female pronuclei disappearance (PNd), and the time to first division (2‐cell). The time point of compaction was considered to be when blastomeres tightened at the morula stage (morula) and individual cell boundaries were considered to be unclear. Start to blastulation (SB) was used to describe the onset of cavity formation. The time point of full blastocyst formation (FB) was defined as the presence of a formed blastocoel that increased in volume and filled the embryo completely.

### 2.9 Statistical analysis

Statistical analyses were performed using GraphPad PRISM 6.03 software (GraphPad Inc., San Diego, CA, USA). Fisher's exact probability test or the chi‐square test was used to evaluate differences in 2‐cell and blastocyst rates. Means in the three groups were compared using one‐way ANOVA and the Tukey‐Kramer test. Differences were considered significant at *p* < 0.05.

## 3 RESULTS

### 3.1 Effect of recovery culture time on the in vitro embryo development of vitrified‐warmed oocytes after ICSI

**FIGURE 1:**
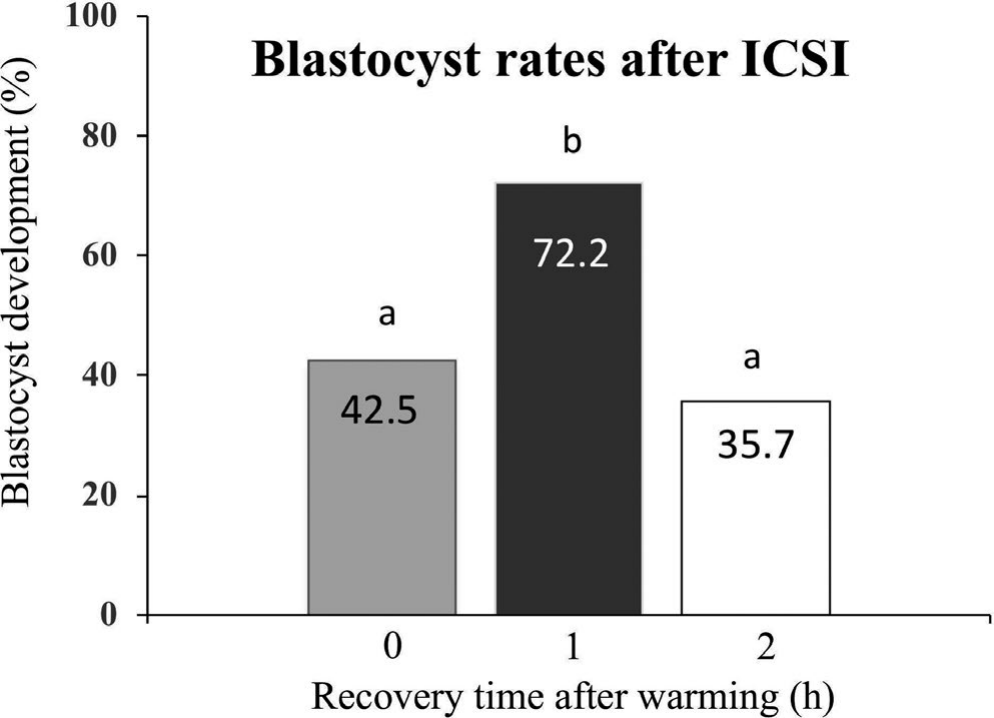
Effect of recovery culture time of vitrified‐warmed mouse oocytes on blastocyst rates following ICSI. ^ab^ *p* < 0.05 (0 vs. 1 or 2 h, chi‐square test)

First, in vitro development of vitrified‐warmed oocytes after ICSI at each time (0, 1, and 2 h) was examined to determine the optimal recovery time from warming to ICSI. As shown in Figure 1, when the vitrified‐warmed oocytes were cultured for 1 h in GSH non‐content recovery medium (global total), the blastocyst rate after ICSI was significantly higher than that after 0 (immediate) or 2 h of recovery culture (72.2% vs. 42.5% and 35.7%, respectively, *p* < 0.05). Therefore, the optimal recovery time from warming to ICSI was decided to be 1 h.

### 3.2 Effect of adding GSH‐OEt to the recovery medium of vitrified‐warmed oocytes on their in vitro development to blastocysts after ICSI

The effects of adding GSH‐OEt to the recovery medium on in vitro embryo development to the 2‐cell and blastocyst stages are shown in Table 1. There was no significant difference in survival rates after warming in all experimental group (100% in 0.5, 1.0, and 3.0 mM GSH‐OEt, respectively). The rates of 2‐cell embryo were significantly higher in 0.5 and 1.0 mM GSH‐OEt than 3.0 mM GSH‐OEt group (89.2, 88.9, and 59.5% respectively; *p* < 0.01). However, the rates of blastocyst formation in the group treated with GSH‐OEt did not differ significantly from those in the vitrified‐warmed group without GSH‐OEt (75.8, 71.9, and 63.6% in 0.5, 1.0, and 3.0 mM GSH‐OEt, respectively). Compared with control fresh oocytes, the 2‐cell and blastocyst rates in vitrified‐warmed oocytes after ICSI were significantly lower despite the presence or absence of GSH‐OEt. The concentration optimization was selected from the results of 2‐cell and blastocyst rates for each concentration. Although there was no statistically significant difference, we selected 0.5 mM because the blastocyst rate was high (75.8%).

**TABLE 1:** Effect of adding of GSH‐OEt to the recovery medium of vitrified‐warmed oocytes on their in vitro development to blastocysts after ICSI

Conc. of GSH‐OEt (mM)	No. of oocytes sperm‐injected	No. (%) of oocytes that survived after warming	No. (%) of 2‐cell embryos	No. (%) of blastocysts/2‐cell embryos
Control^1^	68	‐	66 (97.1)^a^	58 (87.9)^a^
0	64	64 (100.0)^a^	54 (84.4)^b^	39 (72.2)^b^
0.5	37	37 (100.0)^a^	33 (89.2)^b^	25 (75.8)^b^
1.0	36	36 (100.0)^a^	32 (88.9)^b^	23 (71.9)^b^
3.0	37	37 (100.0)^a^	22 (59.5)^c^	14 (63.6)^b^

Note

Vitrified‐warmed oocytes were cultured in recovery medium with or without GSH‐OEt for 1 h up to ICSI.

For this treatment, 0.5, 1.0, and 3.0 mM GSH‐OEt were added into global total medium.

^a,b^Different superscripts indicate a significant difference (*p* < 0.05), based on Fisher's exact probability test.

^1^ Fresh oocytes were cultured in global total medium without GSH‐OEt for 1 h up to ICSI, used as controls.

### 3.3 Effects of vitrification and warming and of GSH‐OEt (0.5 mM) in the recovery medium on GSH and ROS levels in vitrified‐warmed oocytes

**FIGURE 2:**
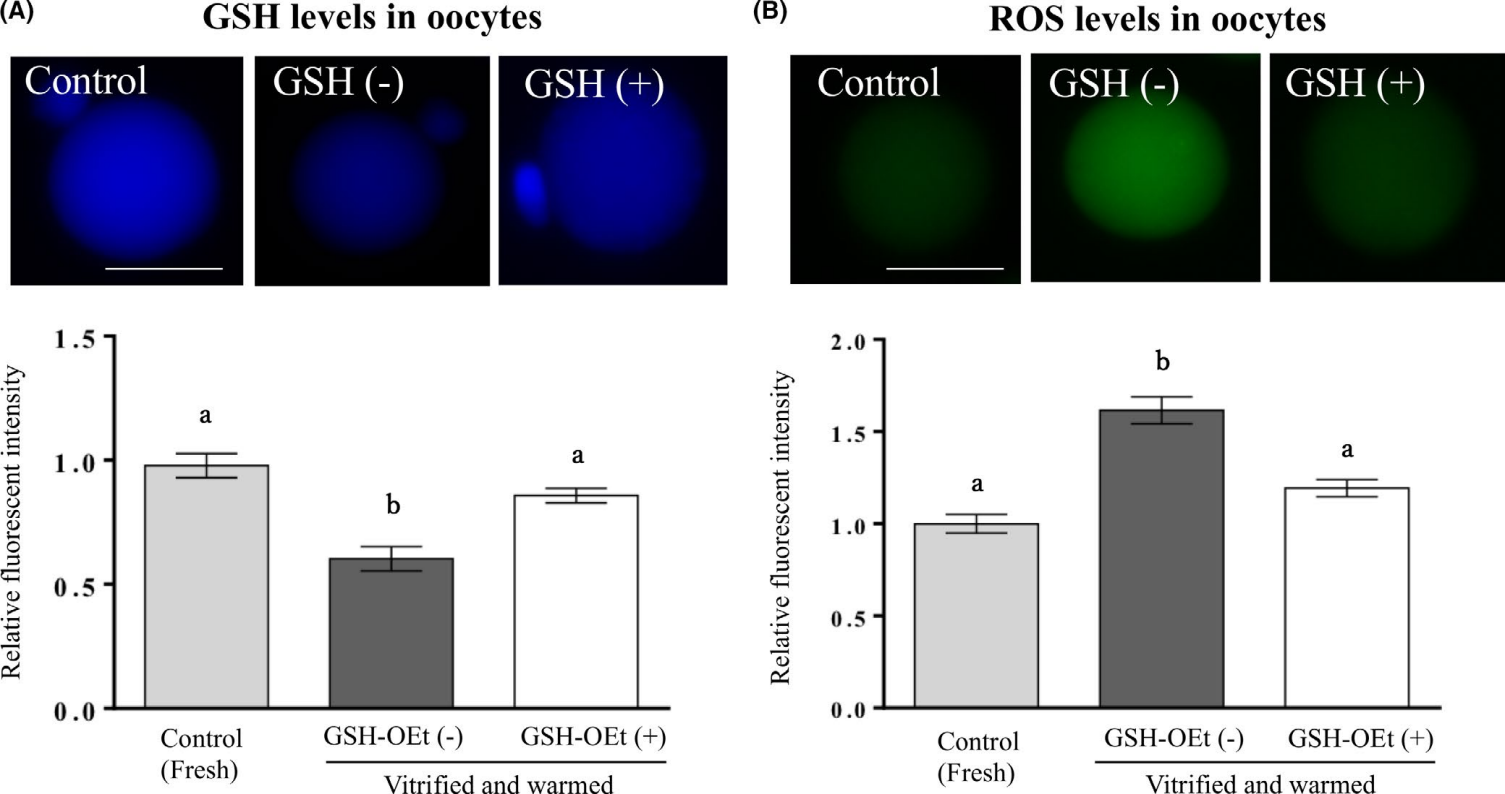
Effect of GSH‐OEt in the recovery medium for 1 h on GSH and ROS levels in vitrified‐warmed mouse oocytes. Fresh oocytes were cultured in global total medium without GSH‐OEt for 1 h, used as controls. In the GSH‐OEt (+) group, GSH‐OEt was added to the global total medium to 0.5 mM and cultured for 1 h. GSH and ROS of oocytes were analyzed using Cell Tracker Blue and CM‐H_2_DCFDA. More than 10 oocytes from each group were analyzed. Each column shows the mean ± SEM. ^ab^ *p* < 0.05, one‐way ANOVA and Tukey‐Kramer test. Bars = 50 μm [Colour figure can be viewed at wileyonlinelibrary.com]

As shown in Figure 2, the GSH level in vitrified‐warmed oocytes was significantly decreased, and the ROS level in vitrified‐warmed oocytes was significantly increased, compared with the GSH and ROS levels in fresh oocytes.

Furthermore, the GSH level in vitrified‐warmed oocytes in the GSH‐OEt (−) group with 1‐h recovery culture without 0.5 mM GSH‐OEt was significantly lower than that in fresh oocytes (Figure 2A). In contrast, the ROS level in vitrified‐warmed oocytes in the GSH‐OEt (−) group was significantly higher than that in fresh oocytes (Figure 2B). When 0.5 mM GSH‐OEt was added to the recovery culture medium for 1 h, the GSH level in the GSH‐OEt (+) group was significantly higher than in the GSH‐OEt (−) group (*p* < 0.05) and was not significantly different from that in the fresh oocytes. That is, after 1‐h recovery culture with GSH‐OEt, the GSH level in vitrified‐warmed oocytes was significantly increased. The ROS level in the GSH‐OEt (+) group was significantly lower than that in the GSH‐OEt (−) group and was not significantly different from that in the fresh oocytes. After 1‐h recovery culture with 0.5 mM GSH‐OEt, the ROS level in vitrified‐warmed oocytes was significantly decreased.

### 3.4 Effect of adding of GSH‐OEt (0.5 mM) to recovery medium of vitrified‐warmed oocytes on their total cell numbers of blastocyst at 96 h from ICSI

Total cell numbers in blastocysts derived from vitrified‐warmed oocytes cultured with or without GSH‐OEt (0.5 mM) in the recovery medium and from fresh control oocytes were counted (Table 2). They were significantly higher in the control and GSH‐OEt (+) groups than in the GSH‐OEt (−) group (93.9 ± 3.9 vs. 94.6 ± 4.5 vs. 73.1 ± 3.1, *p* < 0.01).

**TABLE 2:** Effect of adding of GSH‐OEt to recovery medium of vitrified‐warmed oocytes on their total cell numbers of blastocysts at 96 h from ICSI

Conc. of GSH‐OEt	No. of blastocysts analyzed	Total cell numbers (Mean ± SEM)
Control^1^	56	93.9 ± 3.0^a^
GSH (−)	27	73.1 ± 3.1^b^
GSH (+)	25	94.6 ± 4.5^a^

^a,b^Different superscripts indicate a significant difference (*p* < 0.01). For comparisons between three groups, one‐way ANOVA and Turkey‐Kramer were used.

^1^ Fresh oocytes were cultured in global total medium without GSH‐OEt for 1 h up to ICSI, used as controls. In the GSH‐OEt (+) group, 0.5 mM GSH‐OEt was added to the global total medium for 1 h up to ICSI.

### 3.5 Time‐lapse analysis of developmental timing from ICSI to blastocyst after 1‐h recovery in culture medium with or without GSH‐OEt (0.5 mM)

Table 3 summarizes the results acquired from time‐lapse imaging of development from ICSI to blastocyst. Early and late morphokinetic parameters were assessed as continuous variables for blastocysts. The early embryonic stages (2PB, PNa, and PNd, 2‐cell) were significantly shorter in duration on average in the GSH‐OEt (+) group than in the GSH‐OEt (−) group (2PB: 1.3 vs. 2.2 h, PNa: 3.5 vs. 4.8 h, PNd: 13.2 vs. 14.0 h, and 2‐cell: 15.2 vs. 17.5 h, *p* < 0.01). The later stages of embryonic development (morula, SB, FB) and the overall time to blastocyst formation were of longer duration in the GSH‐OEt (−) group (morula: 57.1 vs. 63.6 h, SB: 73.3 vs. 78.2 h, and FB: 80.5 vs. 86.3 h, *p* < 0.01). Also, in the GSH‐OEt (+) group, the kinetics of blastocyst development were similar to those of the fresh oocyte controls.

**TABLE 3:** Time‐lapse analysis of vitrified and warmed mouse oocytes cultured in the recovery medium with or without GSH‐OEt following ICSI

	Control^1^	GSH‐OEt (−)	GSH‐OEt (+)
No. of oocytes	*n* = 32	*n* = 30	*n* = 31
Developmental stages	Mean (±SD) time to each stage (h)		
2nd polar body (2PB)	1.2 [±0.43]^a^	2.2 [±0.86]^b^	1.3 [±0.46]^a^
Pronuclear appearance (PNa)	3.5 [±0.44]^a^	4.8 [±1.31]^b^	3.5 [±0.62]^a^
Pronuclear disappearance (PNd)	13.2 [±0.79]^a^	14.0 [±4.07]^b^	13.2 [±1.03]^a^
First division (2‐cell)	15.2 [±0.75]^a^	17.5 [±4.01]^b^	15.2 [±1.18]^a^
Morula	57.1 [±3.32]^a^	63.6 [±3.08]^b^	59.1 [±3.85]^a^
Start to blastulation (SB)	72.6 [±3.76]^a^	78.2 [±5.12]^b^	73.3 [±5.63]^a^
Full blastocyst (FB)	79.6 [±3.28]^a^	85.3 [±4.78]^b^	80.5 [±5.40]^a^

^a,b^Different superscripts indicate significant differences between groups at the same developmental stage (*p* < 0.05). For comparisons between the three groups, one‐way ANOVA and the Tukey‐Kramer test were used.

^1^ Fresh oocytes were cultured in global total medium without GSH‐OEt for 1 h up to ICSI, used as controls. In the GSH‐OEt (+) group, 0.5 mM GSH‐OEt was added to the global total medium for 1 h up to ICSI.

## 4 DISCUSSION

Oocyte vitrification is now widely used in human ART. However, whether the ultra‐rapid vitrification method adversely affects oocytes is not yet fully known. In this study, we examined the effect of adding 0.5 mM GSH‐OEt to the recovery medium after vitrification and warming on division kinetics and blastocyst quality in mouse vitrified‐warmed oocytes. Our results show that the addition of 0.5 mM GSH to the recovery medium improved the quality of blastocysts derived from vitrified‐warmed mouse oocytes.

First, we examined the blastocyst rates after ICSI to determine the recovery time after warming. Recovery culture of vitrified‐warmed oocytes for 1 h led to higher blastocyst rates. On the other hand, the blastocyst rate was lower when vitrified‐warmed oocytes were fertilized by ICSI without recovery culture (0 h) or with recovery for 2 h. Generally, recovery culture times of 2–4 h have been used with vitrified‐warmed human oocytes.^7^, ^31^ However, in our study using mouse oocytes, the developmental competence of vitrified‐warmed oocytes worsened at longer times. This result may be closely associated with an increase in intracellular calcium caused by cryoprotectants. EG and DMSO included in the vitrification solution have been shown to cause a rise in mouse oocyte intracellular calcium.^32^ When oocytes are vitrified in calcium‐free vitrification media, the results may differ.

Next, we found that 0.5 mM GSH‐OEt added to the recovery medium of vitrified‐warmed mouse oocytes improved the quality of the blastocysts. Improved development rates in embryos derived from vitrified‐warmed oocytes after IVM in medium supplemented with 1.0 mM GSH‐OEt have been reported in mice.^25^ Treatment with 5 mM GSH‐OEt significantly increases the intracellular GSH content of IVM oocytes and blastocyst total cell numbers in bovine oocytes.^22^ The concentration of GSH‐OEt to be added differs between species, and it is necessary to determine the optimum concentration of GSH‐OEt. The 2‐cell rate in the 3.0 mM GSH‐OEt group was significantly decreased, but the number of blastocysts per 2‐cell embryo was not significantly different from those in the other groups. To explain the decreased developmental competence of vitrified oocytes under the high concentration (3.0 mM) of GSH‐OEt, high levels of intracellular GSH upset may disturb the redox homeostasis in the oocytes and affect the normal fertilization and embryo development. Also, in mouse, adding 1.0 mM GSH, a non‐membrane‐permeable compound to vitrified and warmed medium increased the blastocyst rates, but a higher concentration (2.0 mM) of GSH decreased the blastocyst rates.^33^ Our data further indicate that adding 0.5 mM GSH‐OEt to the recovery medium confers no benefit to blastocyst development. In contrast, there was a significant increase in blastocyst cell numbers when GSH‐OEt was added to the recovery medium, suggesting that the deterioration of oocyte quality due to vitrification is reduced and that blastocyst quality is improved.

We showed that the ROS level in oocytes increased when mouse oocytes were vitrified‐warmed. Furthermore, the GSH level in vitrified‐warmed oocytes was significantly decreased with the increase in ROS. To the best of our knowledge, this is the first study to indicate vitrification decrease GSH level of oocyte. The decrease in intracellular GSH content or alteration in GSH/GSSG redox status may have been induced by increased generation of ROS in vitrification and warming. Embryos cultured in vitro are exposed to a large amount of active oxygen with or without cryopreservation, and this may affect developmental kinetics and embryo development, leading to a decline in embryo quality. Similarly, we speculate that osmotic and thermal stress caused by vitrification, or exposure to high levels of cryoprotectant, may induce a decline in intracellular GSH in MII oocytes. Furthermore, the intracellular GSH concentration decreases significantly as development progresses from the unfertilized oocyte to the blastocyst.^34^ A prior study confirmed that the high intracellular GSH levels in mouse oocytes promote early embryo development,^35^ suggesting that maintaining a high intracellular GSH concentration in unfertilized mouse oocytes is important for embryonic development.

In our time‐lapse study, using ICSI as starting point, the timing from second polar body appearance to time to blastocyst formation was significantly earlier in the 0.5 mM GSH group than in the non‐GSH group, in which it was similar to the control group (fresh oocytes). This result was consistent with previously reported studies in human that the timing from fertilization to blastocyst formation is delayed in embryos derived from vitrified‐warmed oocytes compared with fresh oocytes.^36^, ^37^ Furthermore, in unfertilized hamster oocytes, GSH plays an important role in male pronucleus formation.^38^ Whether there is a relationship between intracellular GSH and sperm swelling in human unfertilized oocytes is not known. However, vitrification can alter the division speed of embryos generated after oocyte warming. A recent study shows the relationship between embryo morphokinetics and metabolic activity.^39^ Vitrification can cause temporary changes in mitochondrial polarity and ATP levels.^40^, ^41^ Further studies are needed to clarify the relationship between these intracellular molecular changes and embryo morphokinetic parameters.

By using a time‐lapse system, the exact timing of each embryo's progress to the blastocyst stage was clarified, and the total cell number of blastocysts was seen to increase. The cell number is an important indicator in assessing the quality of blastocysts. Early arrival at the blastocyst stage increased the blastocyst cell number at that time and improved the quality of the blastocyst.

This study did not clarify the mechanism of action of GSH synthesis and ROS removal upon the addition of GSH‐OEt. Our results suggest that adding GSH‐OEt to the recovery medium can shift the quality of a warmed oocyte closer to the better quality of fresh oocytes, ultimately improving oocyte vitrification outcomes.

In conclusion, our results are evidence that recovery in the presence of GSH‐OEt can enhance the developmental potential of mouse MII oocytes after cryopreservation by vitrification. However, further studies are needed to determine whether GSH‐OEt is also useful in the vitrification of human oocytes.

## CONFLICT OF INTEREST

The authors declare no conflict of interest.

## HUMAN RIGHTS STATEMENTS AND INFORMED CONSENT

This study did not include human participants.

## ANIMAL STUDIES

All the experiments were approved by the Committee for Ethics on Animal Experiments at the Prefectural University of Hiroshima, Japan (18A010).
